# Supplementary material for: Chromothripsis during telomere crisis is independent of NHEJ, and consistent with a replicative origin
Source: Genome Res. 2019 May;29(5):737–49. doi: 10.1101/gr.240705.118 (PMC6499312; doi:10.1101/gr.240705.118)
Supplement: Supplemental Material [file supp_gr.240705.118_Supplemental_file_1.zip › contigs/annotated_contigs/DB91/contig.2.DB91_length_670_mean_cov_10.9014925373.docx]

**DB91_length_670_mean_cov_10.9014925373**

```
TACAATAAATAATCTCCCCTTGTTTGCCTCCATTTGCCCAAAGCCCCCTTCACCCCTTCACGCAGTTAACCACTGTTCCTAGTTTCTTT
 >chr16:68710842-68711178 + E=2e-187
CCTTCCAGAGTTTATATATAGTCTATACTTACATGTGTTACATATTTATTTATATTGTACATGTTTTTACATGTTACCTATTTCGTATA

TATACATATATAAAAACAGATTCTTTTCCTTATGCAAAAGCTAAGATGCTGTTCACATGTCTGCACCTTTCTTTTTCTACTTAACAGTG

TATCAGCACAGCACAATGTCTACCCTCTTTCCACAGATAGCCACTATTAGTTTTTTTCTGTATTCTCC|C|CAGGGACCCCCATCCAAA
                                                                     >chr16:68712217-68
GGCCTGCATGAGATTTCTTGCTACTGAATGTGGGGGCTGAGACAGAAAAACCTCTCCTGTTCAGTGAGCAGATTCTCCTATGGCAGTGC
712552 + E=8e-187
CCCTCTTCACAGAGGACTCTTTGTCACAGTCATGGGATATTTGTTACTTTGTCAGCTGCCATTTTCTTTTCCCTCCAGCTCAAGTCTAA

TAAAGATAGAGACACCAAGATTTTCTACAGCATCACGGGGCCGGGGGCAGACAGCCCCCCTGAGGGTGTCTTCGCTGTAGAGAAGGAGA

CAGGCTGGTTGTTGTTTAATAAGCCACTGGACCGGGAGGAGATTGCCAA
```
